# Current and future treatment strategies in chronic lymphocytic leukemia

**DOI:** 10.1186/s13045-021-01054-w

**Published:** 2021-04-26

**Authors:** Krish Patel, John M. Pagel

**Affiliations:** Center for Blood Disorders and Stem Cell Transplantation, Swedish Cancer Institute, 1221 Madison St, Seattle, WA 98104 USA

**Keywords:** Chronic lymphocytic leukemia, BTK inhibitors, Emerging treatment, Treatment strategy

## Abstract

Treatment decisions for patients with chronic lymphocytic leukemia (CLL) are dependent on symptoms and classification into high-, medium-, or low-risk categories. The prognosis for CLL hinges, in part, on the presence or absence of less-favorable genetic aberrations, including del(17p), del(11q), *TP53* dysfunction, and *IGHV* mutations, as these markers are associated with worse treatment response. Promising results from multiple clinical trials show emerging therapies targeting Burton tyrosine kinase, B-cell leukemia/lymphoma 2, and phosphatidylinositol 4,5-bisphosphate 3-kinase catalytic subunit delta result in better outcomes and prolonged progression-free survival for patients both with and without certain high-risk aberrations. Favorable outcomes using these novel oral targeted therapies, either alone or in combination with other treatments such as anti-CD20 antibodies, has led to their use almost entirely supplanting chemoimmunotherapy in the treatment of CLL. In this narrative review, we summarize the current clinical evidence for the use of targeted mono- and combination therapies for CLL, discuss new and next-generation treatment approaches currently in development, and provide insight into areas of unmet need for the treatment of patients with CLL.

## Background

Chronic lymphocytic leukemia (CLL) is the most common leukemia in Western countries [1] and affects more men than women (a ratio of approximately 1.7:1), with a median age at diagnosis of 67–72 years [2–4]. As a disease of neoplastic mature clonal B lymphocytes, B-cell receptor signaling plays an important role in the survival of CLL cells [5]. Typical clinical signs of active CLL include anemia, thrombocytopenia, and lymphocytosis; symptoms include unintentional weight loss, fatigue, fevers, and drenching night sweats [1, 6]. Overall survival (OS) of patients with CLL at 5 years ranges from about 20% among very high-risk patients to more than 90% in those with less-aggressive genetic risk features [7]. CLL and small lymphocytic lymphoma (SLL) are different clinical presentations of the same pathologic disease and are commonly referred to together as CLL.

Novel, oral, targeted therapies have almost entirely supplanted chemoimmunotherapy in the treatment of CLL. These novel therapies include inhibitors of Bruton tyrosine kinase (BTK), apoptosis regulator B-cell leukemia/lymphoma 2 (BCL-2), and phosphatidylinositol 4,5-bisphosphate 3-kinase catalytic subunit delta (PI3Kδ).

In addition to monotherapy with these oral targeted agents, combinations with other types of therapies are also common. One example is combination therapy with select targeted agents and anti-CD20 monoclonal antibodies (eg, rituximab, ofatumumab, or obinutuzumab) [8]. Emerging therapies include novel chemotherapy-free triplet combinations, bispecific antibody-based, and cell-based therapies for CLL [9–11]. Here we review current and potential future treatment strategies for patients with CLL and explain the underlying rationales, with a focus on real-world evidence.

## Decision to treat

The decision to start treatment of CLL depends on the patient’s symptoms and risk of disease progression [6]. Low-, intermediate-, or high-risk is determined using the widely accepted Rai and Binet clinical-staging systems [12, 13]. Additionally, the CLL international prognostic index (CLL-IPI) combines clinical staging with age, the presence of leukemia cells with *TP53* aberrations or unmutated *IGHV*, and serum β2-microglobulin levels [7]. Treatment is generally indicated in patients with symptomatic or active disease, but not typically among those with no symptoms, as set out in the International Workshop on CLL (iwCLL) guidelines [6]. Prognostic markers are important to consider when deciding how to treat a patient. Less-favorable CLL prognostic markers such as del(17p) or *TP53* dysfunction do not lead to long-term remissions with standard chemoimmunotherapy and patients with these disease features are best treated with novel agents [14].

### Consideration of health-related quality of life

Optimizing health-related quality of life (HRQoL) and managing adverse events (AEs) are also important considerations in the decision to treat and choice of therapy. A 2016 systematic literature review found that greater disease severity was a predictor of poor HRQoL [15]. Comparisons of treated and untreated populations demonstrated small positive effects on HRQoL in favor of treated populations, but HRQoL differences between the treatments were small [15]. However, HRQoL may affect treatment adherence [16]. A retrospective US commercial claims database study reported that the number of AEs experienced by a patient was a significant predictor of poor adherence to oral targeted therapies for hematologic malignancies [17]. In addition to affecting HRQoL, AEs can lead to increased economic burden. The economic burden of AEs in patients receiving CLL therapy was reported to be substantial in a retrospective insurance database study [18]. Because of the toxicity associated with continuous long-term targeted therapy, consideration should be given to the use of limited-duration combination therapies when appropriate [19].

### Early intervention

A phase 3 trial (NCT02863718) was recently conducted to evaluate early-stage treatment of CLL in patients with disease that was not indicated for treatment according to iwCLL guidelines [20]. Patients with Binet stage A disease (with intermediate, high, or very high risk of disease progression) were treated with ibrutinib or placebo. Event-free survival was 47.8 months in the placebo group and not reached in the ibrutinib group at a median follow-up of 31 months; progression-free survival (PFS) was 14.8 months and not reached, respectively. Incidence rates of AEs were similar in both groups. This study demonstrates that early intervention can provide clinical benefit to patients with CLL.

## Current treatment strategies

### BTK inhibitors

BTK inhibitors have become a recommended first-line treatment option in patients with CLL, whether or not they have *TP53* dysfunction, and whether or not their disease has relapsed or become refractory on other treatments [8]. BTK-inhibitor monotherapy is associated with remarkable single-agent efficacy and favorable toxicity compared with chemoimmunotherapy [21]. BTK inhibitors approved for treatment of CLL at the time of writing are ibrutinib and acalabrutinib; others are currently in development (discussed in the “Future treatment strategies” section).

Despite the current treatment guidelines and notable efficacy with BTK inhibitors, an interim analysis from a prospective observational registry study (informCLL) indicated that, in the real-world setting, the transition to BTK inhibitor use according to treatment guidelines has been slow [22]. The study reported that, in 2020, chemoimmunotherapy was still the most common first-line therapy for treatment-naïve patients and that the BTK inhibitor, ibrutinib, was the most commonly used among patients being treated for relapsed or refractory CLL [22]. The informCLL registry also showed that many patients with *TP53* dysfunction received chemotherapy in the real-world setting [22], despite treatment guidelines recommending against chemotherapy in these patients because of the primary ineffectiveness of chemotherapy in this subgroup [6, 8]. Moreover, prognostic genetic testing is required to determine whether *TP53* dysfunction is present, but testing rates were reportedly low [22], which likely accounts for some patients being treated differently from treatment-guideline recommendations.

Patients who were considered high-risk because of del(17p), del(11q), unmutated *IGHV*, or *TP53* dysfunction when chemotherapy was the only available first-line therapy, have much-improved outcomes with BTK-inhibitor therapy. For some risk markers, BTK inhibitors appear to have changed the definition of high risk. For example, when patients were treated with ibrutinib as first-line, those with del(11q) were reported to have a comparable PFS to those without [23], and PFS may be similar whether a patient has CLL with unmutated or mutated *IGHV* [24]. However, del(17p) and *TP53* mutation or deletion remains a risk factor for disease progression on BTK-inhibitor therapy [24].

Ibrutinib was the first BTK inhibitor investigated for the treatment of patients with CLL. Approval for the treatment of patients with relapsed or refractory CLL was obtained in February 2014 [25] and, in March 2016, approval was obtained for treatment-naïve patients with CLL [26]. As of 2019, ibrutinib has been recommended as an option for first-line therapy for all patients with CLL under the National Comprehensive Cancer Network guidelines [27].

The efficacy of ibrutinib compared with chemoimmunotherapy has been established in randomized controlled trials in various settings. The findings from these studies are summarized in Table 1. Briefly, the RESONATE trial showed improved PFS, OS, and overall response rate (ORR) for ibrutinib versus ofatumumab in patients with previously treated CLL [28]. In treatment-naïve patients, ibrutinib was superior to chlorambucil in the RESONATE-2 trial (PFS, OS, and ORR) [29] and the iLLUMINATE trial showed significantly improved PFS with ibrutinib plus obinutuzumab versus chlorambucil plus obinutuzumab [30]. Improved PFS and OS were reported for ibrutinib plus rituximab versus fludarabine plus cyclophosphamide plus rituximab for fit treatment-naïve patients with CLL who were under the age of 70 years in the ECOG1912 trial [31]. Ibrutinib with or without rituximab also improved PFS (but not OS at short follow-up) versus bendamustine plus rituximab in older (≥ 65 years) treatment-naïve patients with CLL (ALLIANCE trial); importantly, the addition of rituximab to ibrutinib did not provide any additional benefit versus ibrutinib alone [32].

**Table 1:** Key clinical trials of approved and investigational BTK inhibitors for the treatment of CLL

Agent	Trial name	Trial design and patients	Outcomes	Citation
*Approved agents*				
Ibrutinib (second-line or later monotherapy)	RESONATE (NCT01578707)	Phase 3 study of the efficacy and safety of ibrutinib versus ofatumumab in patients with relapsed or refractory CLL	Ibrutinib versus ofatumumab: Median PFS (9.4-month median follow up): not reached versus 8.1 months 12-month OS: 90% versus 81% ORR: 42.6% versus 4.1% Grade ≥ 3 AEs: 57% versus 47%	[28]
Ibrutinib (first-line monotherapy)	RESONATE-2 (NCT01722487)	Phase 3 study of the efficacy and safety of ibrutinib versus chlorambucil in treatment-naïve patients (≥ 65 years of age) with CLL	Ibrutinib versus chlorambucil: Median PFS (18.4-month median follow up): not reached versus 18.9 months 24-month OS: 98% versus 85% ORR: 86% versus 35%	[29]
Ibrutinib (first-line combination)	iLLUMINATE (NCT02264574)	Phase 3 study of the efficacy and safety of ibrutinib plus obinutuzumab versus chlorambucil plus obinutuzumab in treatment-naïve patients with CLL/SLL	Ibrutinib plus obinutuzumab versus chlorambucil plus obinutuzumab: Median PFS (31.3-month median follow up): not reached versus 19.0 months 30-month PFS: 79% versus 31% SAEs: 58% versus 35%	[30]
Ibrutinib (first-line combination)	ECOG1912 (NCT02048813)	Phase 3 study of the efficacy and safety of ibrutinib plus rituximab or fludarabine plus cyclophosphamide plus rituximab (chemo-immunotherapy) in treatment-naïve patients (≤ 70 years of age) with CLL	Ibrutinib plus rituximab versus fludarabine plus cyclophosphamide plus rituximab: 3-year PFS: 89.4% versus 72.9% 3-year OS: 98.8% versus 91.5% Subgroup analysis of patients without *IGHV* mutation: 3-year PFS: 90.7% versus 62.5% Grade ≥ 3 AEs: 80.1% versus 79.7%	[31]
Ibrutinib (first-line monotherapy or combination)	ALLIANCE (NCT01722487)	Phase 3 study of the efficacy of ibrutinib, ibrutinib plus rituximab, or bendamustine plus rituximab in treatment-naïve patients (≥ 65 years of age) with CLL	Ibrutinib, ibrutinib plus rituximab, bendamustine plus rituximab: Median PFS: not reached, not reached, 43 months 2-year PFS: 87%, 88%, 74% No differences in OS Grade ≥ 3 hematological AEs: 41%, 39%, 61%	[32]
Acalabrutinib (second-line or later monotherapy)	NCT02029443	Phase 1b/2 study of the safety and efficacy of acalabrutinib in patients with relapsed CLL	Median PFS (41-month median follow up): not reached Median duration of response: not reached 45-month estimated PFS: 62% ORR: 94% Responses were similar regardless of the presence of del(11q), del(17p), complex karyotype, of *IGHV* mutation status Most AEs were mild or moderate	[41, 42]
Acalabrutinib (first-line monotherapy or combination therapy)	ELEVATE TN (NCT02475681)	Phase 3 study of the efficacy and safety of acalabrutinib or acalabrutinib plus obinutuzumab versus chlorambucil plus obinutuzumab in treatment-naïve patients with CLL	Acalabrutinib, acalabrutinib plus obinutuzumab, chlorambucil plus obinutuzumab: Median PFS (28.3-month median follow up): not reached, not reached, 22.6 months Estimated 24-month PFS: 87%, 93%, 47% Consistent across subgroups, including del(17p) Median OS: not reached in any arm Estimated 24-month OS: 95%, 95%, 92% ORR: 86%, 94%, 79%	[43]
Acalabrutinib (second-line or later monotherapy)	ASCEND (NCT02970318)	Phase 3 study of the efficacy and safety of acalabrutinib versus rituximab plus idelalisib or bendamustine in patients with relapsing or refractory CLL	Interim analysis (acalabrutinib versus rituximab plus idelalisib/bendamustine): Median PFS (16.1-month median follow-up): not reached versus 16.5 months 12-month PFS rate: 88% versus 68% Improvement was seen across subgroups, including del(17p) and *TP53* mutation 12-month OS: 94% versus 91% ORR: 81% versus 75%	[44]
*Investigational agents*				
Zanubrutinib (second-line or later monotherapy)	NCT02343120	Phase 1 study of the safety and efficacy of zanubrutinib in patients with CLL	ORR: 96% Estimated 12-month PFS rate: 100% Most AEs were grade 1/2	[38]
Zanubrutinib (first-line monotherapy [Arm C])	SEQUOIA (NCT03336333)	Phase 3 study of zanubrutinib versus bendamustine plus rituximab in treatment-naïve patients with CLL/SLL	Interim analysis (Arm C: del(17p)): Median PFS (18.2 month median follow-up): not reached Estimated 18-month PFS: 89% Median duration of response: not reached Estimated 18-month duration of response: 84% Median OS: not reached Estimated 18-month OS: 95% ORR: 95% Low rate of discontinuation due to AEs	[76]
Zanubrutinib (second-line or later monotherapy)	ALPINE (NCT03734016)	Phase 3 study of zanubrutinib versus ibrutinib in patients with relapsing or refractory CLL/SLL	Trial is ongoing	[74]
Orelabrutinib (second-line or later monotherapy)		Phase 2 study of orelabrutinib in Chinese patients with relapsing or refractory CLL/SLL	Median PFS (median follow-up 14.3 months): not reached Estimated 12-month PFS: 81.1% Median duration of response: not reached Estimated 12-month duration of response: 77.1% Estimated 12-month OS: 86.3% ORR (≥ 12 cycles): 91.3% del(17p): 100% del(11q): 94.7% *TP53* mutation: 100% Unmutated *IGHV*: 93.9% Most AEs were mild to moderate	[79]
LOXO-305 (any-line monotherapy)	BRUIN (NCT03740529)	Phase 1/2 study of LOXO-305 in treatment-naïve patients or those with previously treated CLL or NHL	Interim analysis (patients with CLL): ORR: 57% among 65 efficacy-evaluable patients ORR with ≥ 6 months follow-up: 77% Median follow-up: 3 months for all patients; 6.7 months for responders	[81]
ARQ 531 (second-line or later monotherapy)	NCT03162536	Phase 1/2 study of ARQ 531 in patients with relapsing or refractory hematologic malignancies (including CLL)	Trial is ongoing	

*AE* adverse event, *BTK* Bruton tyrosine kinase, *CLL* chronic lymphocytic lymphoma, *IGHV* immunoglobulin heavy chain, *NHL* non-Hodgkin lymphoma, *ORR* overall response rate, *OS* overall survival, *PFS* progression-free survival, *SAE* serious adverse event, *SLL* small lymphocytic lymphoma

While ibrutinib is generally well-tolerated, treatment discontinuations or interruptions due to toxicity may limit the efficacy of ibrutinib in patients receiving continuous oral therapy. A recent review reported that discontinuation rates for ibrutinib were similar between clinical trials and real-world practice (32% vs 34%, respectively), though the reasons for discontinuation differed [33]. One large-scale, real-world study reported that 41% of patients discontinued ibrutinib and that ibrutinib toxicity was the main reason for these discontinuations [34]. A single-center, real-world study reported that 24% of patients discontinued ibrutinib due to serious adverse events (SAEs), and 55% of patients had a dose interruption of at least 1 week [35]. Temporary ibrutinib interruption was associated with shorter event-free survival in a retrospective study of patients treated outside of clinical trials at the Mayo Clinic [36] and a post hoc analysis of two phase 3 studies (RESONATE and RESONATE-2) found that outcomes after ibrutinib discontinuation were better in patients who received ibrutinib in earlier rather than later lines of therapy [37]. In addition to possibly affecting outcomes, AEs can affect patient willingness to adhere to treatment.

One aspect identified for improvement with BTK inhibitors is to reduce drug-associated toxicities. BTK inhibitors have varying affinities for related and unrelated ATP-binding kinases that contain sterically available cysteines, including epidermal growth factor receptor (EGFR), human EGFR-2 (HER2), human EGFR-4 (HER4), interleukin-2–inducible T-cell kinase (ITK), bone marrow tyrosine kinase gene in chromosome X (BMX), Janus kinase 2 (JAK2), TEC protein tyrosine kinase, and B-lymphocyte kinase (BLK) [38]. Off-target inhibition by these kinases may contribute to many of the toxicities associated with these agents [38], making BTK inhibitors with a higher selectivity potentially more attractive than those with a lower selectivity.

The BTK inhibitor arsenal has expanded recently with the November 2019 approval of acalabrutinib for the treatment of adult patients with CLL [39]. Acalabrutinib is a second-generation BTK inhibitor with reduced off-target activity and improved in vitro selectivity compared with ibrutinib [40]. This greater selectivity for BTK may result in improved efficacy and tolerability. Another BTK inhibitor, zanubrutinib, is currently in development and is discussed later in this review (“BTK inhibitors in development” section); however, it is noted here that, like acalabrutinib, zanubrutinib appears to have greater selectivity than ibrutinib.

Multiple clinical trials in various clinical settings have been conducted to assess the safety and efficacy of acalabrutinib for the treatment of CLL; key studies are summarized in Table 1. The first phase 1/2 study in patients with relapsed CLL demonstrated that acalabrutinib treatment was well-tolerated [41]. After a median follow-up of 41 months, median PFS was not reached, and acalabrutinib showed favorable safety, response, and durability of response; of note, responses were similar among all patients, including those with del(17p), *TP53* dysfunction, unmutated *IGHV,* del(11q), or complex karyotype [42]. The pivotal clinical trial (ELEVATE TN) compared acalabrutinib monotherapy or acalabrutinib plus obinutuzumab to chlorambucil plus obinutuzumab in treatment-naïve patients with CLL who were over the age of 65 years or who had significant comorbidities [43]. In this trial, acalabrutinib demonstrated an acceptable safety profile and significantly improved PFS (for both acalabrutinib and acalabrutinib plus obinutuzumab) versus chlorambucil plus obinutuzumab, which was consistent across subgroups, including patients with high-risk genetic aberrations [43]. Further, interim analysis of the ASCEND phase 3 study demonstrated superiority of acalabrutinib to rituximab plus idelalisib/bendamustine in prolonging PFS in patients with relapsing or refractory CLL, including those with del(17p) or *TP53* mutation or deletion, and showed acalabrutinib to have a more tolerable safety profile [44].

Acalabrutinib has also shown acceptable tolerability in patients who were intolerant to ibrutinib [40, 45], providing an option for continuing BTK-inhibitor therapy in these patients. Most recently, a not-yet published clinical trial, ELEVATE RR, has been completed comparing acalabrutinib directly with ibrutinib in patients with previously treated high-risk CLL (NCT02477696). The results of this trial may have the potential to change clinical practice in favor of acalabrutinib.

As with most cancer therapies, resistance to treatment can develop. In the case of current-generation BTK inhibitors, acquired mutations in *BTK* that affect the active site or are immediately downstream of the effector phospholipase C γ2 (*PLCG2*) can lead to BTK-inhibitor resistance and relapse in patients with CLL [46, 47]. Therefore, there is a need to identify and develop next-line therapies for these patients who develop BTK-inhibitor resistance and to understand how to identify the optimal treatment sequence for each individual patient.

### Inhibitors of BCL-2 and PI3Kδ

#### BCL-2 inhibitor

Other approved drug classes for treating CLL include BCL-2 and PI3Kδ inhibitors; key clinical trials investigating their efficacy and safety are summarized in Table 2 (BCL-2 inhibitors) and Table 3 (PI3Kδ inhibitors). Venetoclax was the first, and remains the only, approved BCL-2 inhibitor for the treatment of patients with relapsed high-risk CLL. It was initially approved in April 2016 for the treatment of patients with del(17p) CLL who had received at least 1 prior line of therapy [48]. Approval was based on the results of the pivotal phase 2 study, which was conducted in patients with del(17p) CLL [49, 50]. Subsequently, the combination of venetoclax plus obinutuzumab for a fixed duration was approved for treatment-naïve patients with CLL in May 2019 [48] based on the results of the CLL14 trial [51]. This study reported that venetoclax plus obinutuzumab given for just 1 year significantly prolonged PFS versus chlorambucil plus obinutuzumab, including in those patients with del(17p), *TP53* mutation or deletion, or unmutated *IGHV* status [51]. Given the current treatment landscape, venetoclax plus obinutuzumab has emerged as another important first-line treatment option, as well as the most obvious first-line treatment, for patients with *TP53* dysfunction and unmutated *IGHV* who are not suitable candidates for BTK-inhibitor monotherapy. It should be noted that follow-up efficacy results from the CLL14 trial, however, suggest that outcomes in patients having disease with *TP53* mutation or deletion may not be as durable, with relatively earlier relapses after therapy discontinuation [52]. A recent real-world study of patients with CLL who were treated with venetoclax identified *TP53* aberrations as a predictor of inferior PFS [53].

**Table 2:** Key clinical trials of approved BCL-2 inhibitors for the treatment of CLL

Agent	Trial name	Trial design and patients	Outcomes	Citation
Venetoclax (second-line or later monotherapy)	NCT01889186	Phase 2 single-arm study of the safety and efficacy of venetoclax in patients with del(17p) relapsed or refractory CLL	ORR: 12.1-month median follow-up, 79.4%; final, 77% 24-month PFS: 54% SAEs: 12.1-month median follow-up, 55%; final, 58%	[49, 50]
Venetoclax (first-line combination therapy)	CLL14 (NCT02242942)	Phase 3 study of the efficacy and safety of venetoclax plus obinutuzumab versus chlorambucil plus obinutuzumab in treatment-naïve patients with CLL who have coexisting conditions	Venetoclax plus obinutuzumab versus chlorambucil plus obinutuzumab: 24-month PFS: 88.2% versus 64.1% Results were similar for *TP53* deletion/mutation or unmutated *IGHV* Grade ≥ 3 AEs: 78.8% versus 76.6%	[51]
Venetoclax (second-line or greater combination therapy)	MURANO (NCT02005471)	Phase 3 study of the efficacy and safety of venetoclax plus rituximab versus bendamustine plus rituximab in patients with relapsed or refractory CLL/SLL	Venetoclax plus rituximab versus bendamustine plus rituximab: 24-month PFS: 84.9% versus 36.3% Results were similar for *TP53* deletion or unmutated *IGHV* Grade ≥ 3 AEs: 82.0% versus 70.2%	[50]

*AE* adverse event, *BCL-2* B-cell lymphoma 2, *CLL* chronic lymphocytic lymphoma, *IGHV*, immunoglobulin heavy chain, *ORR* overall response rate, *PFS* progression-free survival, *SAE* serious adverse event, *SLL* small lymphocytic lymphoma

**Table 3:** Key clinical trials of approved and investigational PI3Kδ inhibitors for the treatment of CLL

Agent	Trial name	Trial design and patients	Outcomes	Citation
*Approved agents*				
Idelalisib (second-line or later combination therapy)	NCT01539512	Phase 3 study of the efficacy and safety of idelalisib plus rituximab versus placebo plus rituximab in patients with relapsed CLL and significant coexisting medical conditions	Idelalisib plus rituximab versus placebo plus rituximab: Median PFS: not reached versus 5.5 months 12-month OS: 92% versus 80% ORR: 81% versus 13% SAEs: 40% versus 35%	[56]
Idelalisib (first-line monotherapy followed by combination therapy)	NCT02135133	Phase 2 study to evaluate idelalisib as first-line therapy; patients were treated with idelalisib monotherapy for 2 months then switched to combination therapy of idelalisib plus ofatumumab for an additional 6 months	Safety data after a median follow-up of 14.7 months: Hepatotoxicity was reported as a frequent, and often severe, AE 79% of patients experienced grade ≥ 1 ALT or AST elevation 54% of patients experienced grade ≥ 3 transaminitis (median time to development: 28 days) Younger age and mutated *IGHV* status were found to be significant risk factors for developing hepatotoxicity The development of idelalisib-related toxicities was reported to be associated with increased levels of inflammatory cytokines and decreased levels of regulatory T cells	[57]
Idelalisib (second-line or later combination therapy)	NCT01659021	Phase 3 study of the efficacy and safety of idelalisib plus ofatumumab versus ofatumumab monotherapy in patients with relapsed CLL	Idelalisib plus ofatumumab versus ofatumumab monotherapy: Median PFS: 16.3 versus 8.0 months Serious infections were more common in the idelalisib plus ofatumumab group versus the ofatumumab monotherapy group Pneumonia: 13% versus 10% Sepsis: 6% versus 1% *Pneumocystis jirovecii* pneumonia: 5% versus 1% Treatment-related deaths: 22 versus 6	[59]
Idelalisib (second-line or later combination followed by monotherapy)	NCT01539291	Phase 3 study of the long-term efficacy and safety of idelalisib plus rituximab followed by idelalisib monotherapy (extension of NCT01659021) in patients with relapsed CLL	Median PFS (18-month median follow-up): 20.3 months Median OS: 40.6 months ORR: 85.5% Prolonged exposure to idelalisib resulted in an increased incidence of AEs including diarrhea, colitis, and pneumonitis; the incidence of elevated hepatic aminotransferases was not increased	[60]
Idelalisib (second-line or later combination therapy)	NCT01569295	Phase 3 study of the efficacy and safety of idelalisib plus bendamustine plus rituximab versus placebo plus bendamustine plus rituximab in patients with relapsed or refractory CLL	Idelalisib plus bendamustine plus rituximab versus placebo plus bendamustine plus rituximab: Median PFS (14-month median follow-up): 20.8 versus 11.1 months All-grade AEs of infections and infestations: 69% versus 59% Grade ≥ 3 AEs of infections and infestations: 39% versus 25%	[61]
Duvelisib (second-line or greater monotherapy)	DUO (NCT02004522)	Phase 3 study of the efficacy and safety of duvelisib versus ofatumumab monotherapy in patients with relapsed or refractory CLL	Duvelisib versus ofatumumab: Median PFS: 13.3 versus 9.9 months ORR: 74% versus 45% PFS and ORR was similar for patients with del(17p)/*TP53* mutations Grade ≥ 3 AEs: 87% versus 48% Infectious AEs: 69% versus 43%	[64]
Duvelisib (second-line or later monotherapy)	NCT02049515	Phase 3 study of the efficacy and safety of duvelisib in patients with relapsed or refractory CLL who were enrolled in the DUO trial and progressed after ofatumumab (DUO extension)	After crossover versus before crossover (to duvelisib): Median PFS: 15.7 and 9.4 months ORR: 77% versus 29% Median duration of response: 14.9 versus 10.4 months Responses were similar for patients with del(17p) and/or *TP53* mutation 73% of patients with disease refractory to ofatumumab achieved a response on duvelisib Safety profile was manageable	[65]
*Investigational agents*				
Umbralisib (second-line or later monotherapy)	NCT02742090	Phase 2 study of umbralisib in patients with CLL who are intolerant of kinase inhibitor therapy	Median PFS: 23.5 months 12% discontinuations due to AEs	[84]
Umbralisib (second-line or later combination therapy)	NCT02006485	Phase 1 study of triplet treatment (umbralisib, ublituximab, and ibrutinib) in patients with advanced B-cell malignancies	ORR: 84% Tolerable safety profile	[86]
Umbralisib (second-line or later combination therapy)	NCT02268851	Phase 1/1b study of umbralisib plus ibrutinib in patients with CLL or mantle cell lymphoma	Interim analysis: Most frequent AEs were diarrhea (52%), infection (50%) and transaminitis (24%) SAEs occurred in 29% of patients	[85]
ME-401 (second-line or later monotherapy or combination therapy)	NCT02914938	Phase 1 study of ME-401 alone, in combination with rituximab, or in combination with zanubrutinib in patients with relapsing or refractory CLL/SLL or B cell NHL	Interim analysis (patients with CLL/SLL [n = 10] treated with monotherapy or combination with rituximab): Median PFS: not reached (median follow-up: 9.7 months) Median duration of response: not reached ORR: 89% (monotherapy: 100%; combination with rituximab: 83%) No apparent safety differences between monotherapy or ME-401 plus rituximab treatment	[91]

*AE* adverse event, *ALT* alanine aminotransferase, *AST* aspartate aminotransferase, *CLL* chronic lymphocytic lymphoma, *IGHV* immunoglobulin heavy chain, *ORR* overall response rate, *OS* overall survival, *PFS* progression-free survival, *PI3K* phosphoinositide 3-kinase, *SAE* serious adverse event, *SLL* small lymphocytic lymphoma

Promising results have also been obtained for treatment of relapsed patients with 2 years of fixed-duration therapy with venetoclax plus rituximab. The MURANO trial reported that this combination resulted in a significantly longer PFS versus bendamustine plus rituximab in patients with relapsed or refractory CLL, regardless of *TP53* or *IGHV* mutation status [50].

It should be noted that venetoclax regimens are generally well-tolerated and effective in patients who were previously treated with ibrutinib [53, 54]. A recent real-world study of CLL patients treated with venetoclax reported that 29% of patients discontinued treatment, most commonly due to disease progression, followed by toxicity (mainly hematologic). This study also reported a dose-reduction rate of 21% [53]. Real-world response rates and durations of responses were noted as comparable to clinical-trial data, with most patients maintaining a maximum recommended dose [53].

A treatment discontinuation rate of approximately 6% due to treatment-related toxicity leaves an opportunity for improvement in next-generation BCL-2 inhibitors. In addition, the development of venetoclax resistance remains a concern. Resistance may develop because of mutations in *BCL2*, further highlighting the need to investigate new treatment combinations to determine optimal treatment strategies [21].

#### PI3K inhibitors

There are currently 2 PI3Kδ inhibitors approved for the treatment of CLL. The first of these, idelalisib, was approved for use in combination with rituximab for the treatment of relapsed CLL in 2014 [55]. While idelalisib is highly effective in combination with rituximab [56], its use as first-line therapy was associated with severe immune-mediated hepatoxicities [57, 58], and its use as second-line or later therapy was associated with an increased incidence of serious infections [59, 60] and an increase in the incidence of additional immune-mediated AEs with prolonged exposure [60, 61].

Given the toxicity issues, treatment with idelalisib has been limited in comparison to BTK inhibitor treatment. When idelalisib was compared with ibrutinib as a first-kinase inhibitor treatment in a retrospective real-world study, ibrutinib appeared superior [62]. In the randomized phase 3 ASCEND trial, acalabrutinib was superior to idelalisib plus rituximab among patients with relapsed or refractory CLL [44].

Duvelisib is the second, and most recently approved, PI3Kδ inhibitor for CLL treatment [63]. It is a dual inhibitor of phosphatidylinositol 3-kinases PI3Kδ and PI3Kγ and was approved in December 2018 for the treatment of patients with relapsed or refractory CLL [63]. While it has demonstrated efficacy in CLL, treatment-associated toxicities have been a concern [64, 65]. Ongoing research aims to discover approaches to reduce the occurrence of treatment-related immune-mediated toxicities through combination-therapy studies and alternative dosing regimens [66, 67]. Similar to idelalisib, the toxicities associated with duvelisib treatment have led, in most cases, to consideration for their use after a BTK or BCL-2 inhibitor as a general treatment strategy.

## Future treatment strategies

BTK inhibitors are an effective treatment for patients with CLL; however, there is a need to further improve upon tolerability issues and the development of resistance. AEs associated with BTK-inhibitor treatment reduce both tolerability and HRQoL. While BTK-target selectivity in second-generation BTK inhibitors appears to have reduced off-target effects, they remain susceptible to development of treatment resistance and additional improvement in tolerance is possible.

The development of resistance to irreversible-covalent-binding BTK inhibitors is almost inevitable in most patients with CLL. There is a need to develop treatment strategies or novel therapies that would delay the development of resistance or overcome the issue altogether.

Determining an optimal treatment sequence would likely benefit patient outcomes. As an example, a large, multicenter, retrospective analysis of treatment sequences in patients with CLL was conducted to better understand the optimal treatment sequence for several newer CLL therapies, ibrutinib, idelalisib, and venetoclax [62]. The study found that, in patients who were treated with kinase inhibitors or venetoclax in the setting of prior kinase-inhibitor failure, alternate kinase inhibitors or venetoclax appeared superior to chemoimmunotherapy combinations. The study also reported that ibrutinib appeared superior to idelalisib as the first kinase inhibitor in patients with relapsed CLL. An open-label phase 2 trial assessed venetoclax treatment in patients with relapsed or refractory CLL who had disease progression during or after treatment with ibrutinib [59]. Interim analysis indicated that venetoclax had durable clinical activity, with a median PFS of 24.7 months and 12-month estimates of PFS and OS of 75% and 91%, respectively. A retrospective chart review found that BTK inhibitor therapy for patients with CLL who had disease progression following venetoclax treatment resulted in durable disease control, with a median PFS of 34 months and a median OS of 42 months after BTK inhibitor initiation [68]. These findings highlight the potential importance of treatment sequence on outcomes. With the development of multiple new therapies for CLL, treatment algorithms should be optimized, requiring clinical studies testing different sequencing strategies.

Despite recent advances in CLL therapy, outcomes for patients with *TP53* mutation or deletion remain worse than for those without. For example, while BTK inhibitors have improved outcomes for certain patients who were considered high-risk on chemotherapy, *TP53* aberration is a risk for progression on BTK-inhibitor therapy [24]. Likewise, a recent real-world study reported a significantly shorter PFS in patients with *TP53* deficiency versus without, and identified *TP53* dysfunction as a predictor of inferior PFS [53]. Taken together, these findings suggest that there is a need for new targeted therapies and new treatment approaches in the high-risk patient population.

New therapies and treatment strategies should not only aim to improve tolerability and to overcome the development of resistance, but also to extend remission with duration-limited approaches, regardless of risk factors. Next-generation BTK, BCL-2, and PI3Kδ inhibitors that may address shortcomings related to tolerability and resistance are in development. Research into the development of novel therapies or drug combinations with the goal of offering a finite treatment option is needed. New treatment strategies that include novel drug combinations, such as BCL-2/BTK-inhibitor combinations and chemotherapy-free triplet combinations, or minimal residual disease (MRD)-guided treatment have shown promise in recent and ongoing clinical trials. Novel therapies that aim to extend survival and work towards cures, such as bispecific antibodies and chimeric antigen receptor T-cell (CAR-T) therapy, are also being developed.

### BTK inhibitors in development

Next-generation BTK inhibitors aim to reduce the development of resistance, though their improvements over currently approved BTK inhibitors remain under investigation. Those agents currently in development for CLL include zanubrutinib and orelabrutinib, and the reversible noncovalent-binding inhibitors LOXO-305 and ARQ 531. Key clinical trials of these investigational agents are summarized in Table 1. LOXO-305 and ARQ 531 have shown activity against BTK inhibitor-resistant CLL in preclinical studies [69, 70] and, while neither is currently approved for the treatment of CLL, both have shown promise in patients with acquired resistance to BTK inhibitors [71, 72].

One of the irreversible BTK inhibitors, zanubrutinib, received accelerated approval in the USA (November 2019) for the treatment of adult patients with mantle cell lymphoma who had received at least 1 previous therapy [73]. Zanubrutinib is not currently approved for CLL but is under investigation for this indication [74]. Data regarding the dynamics, number, and immunophenotype of immune cells collected from patients with relapsed or refractory CLL and who were undergoing zanubrutinib treatment suggest that zanubrutinib can regulate immunity by improving T-cell exhaustion, inhibiting suppressor cells, and disrupting CLL cell migration through downregulation of adhesion/homing receptors [75]. A phase 1 study demonstrated that zanubrutinib had favorable tolerability and encouraging activity in patients with CLL [38]. Recent results from Arm C (treatment-naïve patients with del(17p) CLL) of the phase 3 SEQUOIA trial showed a durable response to zanubrutinib at the median follow-up of 18.3 months and that treatment was generally well-tolerated, with a low rate of discontinuation due to AEs [76]. Direct comparisons of the efficacy and safety of zanubrutinib and ibrutinib have been reported in patients with relapsed Wäldenstrom macroglobulinemia (ASPEN trial; NCT03053440) [77] and are currently underway in patients with relapsed or refractory CLL (ALPINE trial; NCT03734016) [74]. Preliminary results from ASPEN reported that the incidence of atrial fibrillation, contusion, diarrhea, peripheral edema, hemorrhage, muscle spasms, pneumonia, and AEs leading to discontinuation or death were lower with zanubrutinib than ibrutinib [78], demonstrating an apparent improved toxicity profile in patients with Wäldenstrom macroglobulinemia. Whether these toxicity differences may exist in patients with CLL is unknown to date, but such questions will be answered by future and ongoing studies, including ALPINE.

Orelabrutinib is a highly selective irreversible BTK inhibitor that is also in development [79] and was recently approved in China for the treatment of patients with relapsed or refractory CLL/SLL and relapsed or refractory mantle cell lymphoma [80]. Orelabrutinib demonstrated durable response in Chinese patients with relapsed or refractory CLL/SLL, including those with del(17p), del(11q), *TP53* mutation, or unmutated *IGHV*, and a favorable safety profile in a recent update of a phase 2 extended study [79].

LOXO-305 is a noncovalent BTK inhibitor that is also under development and currently undergoing a phase 1/2 study (BRUIN trial; NCT03740529) in patients with previously treated CLL or non-Hodgkin lymphoma (NHL). Recent phase 1 data indicated favorable safety and promising efficacy (ORR: 57%) in heavily pretreated patients with CLL, including those with acquired BTK-inhibitor and venetoclax resistance [81]. Follow-up studies will be needed to confirm these results.

Also currently in early-phase clinical trials is ARQ 531, a multikinase inhibitor of BTK- and Src-family kinases. ARQ 531 is presently being evaluated in an open-label, multicenter phase 1/2 trial in patients with a number of B-cell malignancies, including CLL (NCT03162536) [82]. Preclinical data suggest that ARQ 531 may be effective against BTK-resistant CLL and CLL that has undergone Richter transformation [70], as well as against acute myeloid leukemia [83].

As clinical development continues for these next-generation BTK inhibitors, it is hoped that one, or all, may emerge as a treatment option for later-line therapy in patients who have developed resistance to earlier-line therapies such as ibrutinib, acalabrutinib, or venetoclax.

### PI3Kδ inhibitors in development

Given the toxicities observed with currently approved PI3Kδ, new drugs in this class are being developed to reduce associated toxicities. Summaries of key clinical trials of these investigational agents can be found in Table 3. One agent in development is umbralisib, an inhibitor of PI3Kδ and casein kinase I isoform epsilon. A phase 2 trial showed that umbralisib monotherapy was effective in patients with CLL who were intolerant to previous kinase-inhibitor treatment [84]. This was the first study to demonstrate that switching to umbralisib from another kinase inhibitor resulted in improved disease control without the recurrence of kinase-inhibitor intolerance toxicities [84]. This level of safety improvement over currently approved PI3Kδ inhibitors may greatly improve the applicability of this class of drugs to CLL treatment regimens.

Umbralisib has also been tested as combination therapy. A phase 1/1b study of umbralisib in combination with ibrutinib in patients with relapsed or refractory CLL or mantle cell lymphoma found that this treatment combination was well-tolerated and demonstrated activity against disease [85]. This first clinical report of doublet therapy using a BTK inhibitor combined with a PI3Kδ inhibitor indicates that it is a feasible approach, though further studies are warranted. Similarly, an open-label phase 1 study of combination therapy with umbralisib, ublituximab, and ibrutinib found the combination to be tolerable with encouraging activity in advanced CLL and B-cell NHL [86]; however, additional investigation of this chemotherapy-free triplet combination is needed. A recent update of the phase 3 UNITY trial (NCT02612311) reported that treatment with umbralisib plus ublituximab significantly prolonged PFS compared with obinutuzumab plus chlorambucil in patients with CLL who were treatment-naïve or who had relapsed or refractory CLL (median PFS: 31.9 months vs 17.9 months; *p* < 0.0001) [87].

Another PI3Kδ inhibitor in development is the highly selective ME-401; it is being tested as a once-daily oral treatment [88]. Preclinical in vitro data demonstrated that ME-401 had more potent activity against CLL cells compared with idelalisib or ibrutinib [89], and in preclinical animal models it was shown to bind to the target more tightly than idelalisib [88]. A high objective response rate was reported in a dose-escalation/expansion phase 1b clinical trial of patients with B-cell malignancies who were treated with ME-401 either continuously or on an intermittent schedule (NCT02914938) [90, 91]. Importantly, patients on the intermittent schedule (2 months of continuous daily therapy, followed by 7 days of ME-401 delivery, then 3 weeks off treatment, in every 28-day cycle) were observed to have a significantly reduced incidence of immune-mediated AEs of special interest compared with patients on continuous treatment. Updated data (median follow-up 9.7 months) from those patients on the intermittent schedule reported a low rate of grade 3 severity class-related AEs of special interest and a continued high objective response rate [91]. These data demonstrate that advances in treatment schedules may help reduce toxicity without compromising efficacy.

### New approaches in development

#### Novel drug combinations

Combinations of anti-CD20 and targeted therapies have been evaluated. Venetoclax plus obinutuzumab for a fixed treatment duration of 1 year is approved for treatment-naïve patients with CLL [48]. Because acalabrutinib has improved kinase selectivity versus ibrutinib [92], and obinutuzumab appears to have improved antibody-dependent cellular toxicity over rituximab [93, 94], evaluation of this combination was warranted. A recently completed phase 1b study evaluating acalabrutinib plus obinutuzumab therapy in treatment-naïve patients with relapsed or refractory CLL reported high response rates and durable remissions [95]. The ELEVATE-TN trial evaluated acalabrutinib both as monotherapy and in combination with obinutuzumab [43]. Post hoc analysis revealed that better PFS was observed with the combination; however, the study was not powered to determine statistical significance for the comparison of these 2 treatment arms. Further studies are needed to determine if there is an advantage for combination versus monotherapy and to identify the appropriate patient population.

BCL-2 and BTK-inhibitor combinations have shown promise, particularly in high-risk patients. The phase 2 CLARITY study evaluated ibrutinib plus venetoclax in patients with relapsed or refractory CLL [96]. After 12 months of combination treatment, 19 of 53 (36%) patients had MRD-negative bone marrow and 28 of 53 (53%) patients had MRD-negative peripheral blood samples. The depth of MRD reduction improved over time, with 11 of 25 (44%) patients achieving MRD eradication after 24 months of treatment. Another phase 2 trial of ibrutinib plus venetoclax was conducted in treatment-naïve, high-risk, older patients with CLL [97]. The proportion of patients with undetectable MRD (uMRD) increased over time (ie, with an increased number of treatment cycles). Of the 80 patients enrolled, 59 (74%) had a best response of complete remission (CR) or CR with incomplete count recovery (CRi). After 18 treatment cycles, 25 of 26 (96%) patients had CR or CRi and 18 of 26 (69%) had bone marrow uMRD. Responses were seen across all high-risk subgroups and no new safety concerns were reported. Longer-term studies are needed to determine if this combination is feasible as a fixed-duration therapy option. A phase 1 trial to determine optimal dosing of ibrutinib when venetoclax is added for the treatment of patients with CLL who have progressed on ibrutinib monotherapy (NCT03422393) is ongoing. Numerous other clinical trials evaluating various aspects of BCL-2/BTK-inhibitor combination treatment are underway; it is hoped that these trials will result in promising new treatment options for CLL, including potential fixed-duration treatment options.

Another treatment strategy aiming to achieve a fixed-duration treatment time with high rates of deep remission is the use of chemotherapy-free triplet combinations. Phase 2 results from a study evaluating limited duration (14 cycles; 28 days/cycle) treatment with ibrutinib, venetoclax, and obinutuzumab in patients who were treatment-naïve and had relapsed or refractory CLL have been published [98]. The ORR was 84% in treatment-naïve patients and 88% in patients with relapsed or refractory CLL. uMRD (assessed in both blood and bone marrow) was achieved in 67% and 50% of patients, respectively, and treatment was well-tolerated, with 6% of patients discontinuing because of AEs, most of which were hematological in nature. A phase 2 trial of this triplet therapy in treatment-naïve patients with high-risk CLL (CLL2-GIVE) reported encouraging preliminary results, with a CR rate of 59% (24/41 patients) and uMRD in the peripheral blood in 33 patients (81%) [99]. Twenty-two patients discontinued treatment at cycle 15 after achieving uMRD and CR or CRi. Phase 3 trials are currently being conducted to compare ibrutinib plus obinutuzumab with and without venetoclax (NCT03701282 and NCT03737981). NCT03701282 will compare MRD and MRD CR rates between treatments as a secondary outcome measure, and NCT03737981 will include MRD analysis. A phase 3 study evaluating multiple venetoclax-containing experimental arms (plus rituximab, plus obinutuzumab, plus ibrutinib and obinutuzumab) versus chemoimmunotherapy in treatment-naïve patients with CLL who do not have del(17p) or *TP53* mutation is also ongoing (GAIA/CLL13 trial, NCT02950051). Studies evaluating triplet combinations using acalabrutinib rather than ibrutinib are also underway. An ongoing phase 2 trial evaluating limited-duration acalabrutinib, venetoclax, and obinutuzumab triplet therapy in treatment-naïve patients with CLL (NCT03580928) has reported updated results: 100% of patients with at least 16 months of follow-up have responded to treatment, with 43% acheiving CR/CRi and 57% achieving partial response. The primary endpoint of bone marrow uMRD CR was achieved by 31% of patients [100]. Additionally, 84% of patients achieved peripheral blood uMRD and 78% achieved bone marrow uMRD. A phase 3 study to evaluate acalabrutinib plus venetoclax with or without obinutuzumab versus chemoimmunotherapy in treatment-naïve patients with CLL was recently initiated (ACE-CL-311, NCT03836261). Other triplet combinations, such as atezolizumab (anti-PD-L1), obinutuzumab, and venetoclax (NCT02846623) are also being investigated in patients with CLL.

#### MRD-guided treatment approaches

MRD in CLL is determined by the number of leukemic cells detected in either the peripheral blood or bone marrow and uMRD has been most often defined as < 1 CLL cell per 10,000 leukocytes [6]. Multiple randomized clinical trials have shown that MRD status after treatment induction is an independent predictor of survival and PFS (reviewed in [101]), and efforts have been made towards determining the feasibility of using an MRD-guided approach to CLL treatment. A retrospective analysis of patients treated with chemoimmunotherapy found that, among patients who achieved uMRD, those who stopped treatment after 3 cycles had PFS and OS outcomes similar to those who received 6 cycles of therapy [102]. Other trials evaluating different strategies for treatment discontinuation after patients achieve uMRD with CR/CRi have shown promising results. Strategies include reduced dosing based on uMRD achievement [103], terminating treatment after uMRD is confirmed in patients with CR/CRi [104], and limiting treatment duration after becoming MRD negative to the time it took to achieve uMRD [96]. The latter approach is being further evaluated in the ongoing phase 3 FLAIR trial [105]. MRD-guided approaches should take into consideration the impact of specific treatments on MRD predictive value. Additionally, studies investigating the impact of disease biology on the predictive value of MRD are needed to understand which patients will benefit most from MRD-guided treatment [101]. It should be noted that MRD-related outcomes are included in the design of multiple ongoing clinical trials in patients with CLL (eg, NCT03737981 and NCT03701282). Continued MRD analysis in clinical trials may provide valuable information towards determining appropriate MRD-guided treatment protocols.

#### Bispecific antibodies

Unlike monospecific antibodies that bind to a single epitope, bispecific antibodies are able to bind 2 distinct epitopes, allowing dual targeting capabilities [106]. This permits the development of antibodies with novel mechanisms of action, such as bringing 2 cell types together (eg, engaging immune and tumor cells), delivering payloads to target cells, or engaging or blocking 2 different antigens on the same cell [106]. One example of a bispecific antibody that is approved for cancer treatment is the bispecific T-cell engager (BiTE) antibody blinatumomab, which binds both CD19 and CD3 and elicits cytotoxic T lymphocyte (CTL) activity against CD19-expressing tumor cells [9, 107].

The development of bispecific antibodies in the CLL treatment space is currently focused on dual targeting of CD3 and CD20. There are 7 CD3/CD20 antibodies that are currently in phase 1 or 1/2 clinical trials for CLL and/or NHL [108]. Plamotamab (XmAb13676), a bispecific antibody that binds both CD3 and CD20, is currently being tested in a first-in-human phase 1 clinical study in patients with CLL and NHL (NCT02924402). Interim results indicate evidence of clinical activity in heavily pretreated patients with relapsed or refractory CLL or NHL and AEs were generally manageable. This study is ongoing and further data specifically from the CLL patient population are expected.

Results from phase 1 clinical trials of CD3/CD20 bispecific antibodies odronextamab (REGN1979) and mosunetuzumab in other B-cell malignancies have been reported. A phase 1 study reported that treatment with odronextamab resulted in overall responses and durable CRs (relapsed or refractory diffuse large B-cell lymphoma without prior CAR-T therapy: ORR 60%; CR 60%; median duration of CR 9.5 months; relapsed or refractory diffuse large B-cell lymphoma with prior CAR-T therapy: ORR 33%; CR 24%; median duration of CR 4.4 months; relapsed or refractory follicular lymphoma: ORR 93%; CR 75%; median duration of CR 8.1 months) [109]. A phase 1 clinical trial investigating the safety and tolerability of odronextamab in patients with CLL or NHL is ongoing (NCT02290951). In a phase 1/1b clinical trial (NCT02500407), patients with relapsed or refractory follicular lymphoma who received fixed-duration mosunetuzumab monotherapy had an ORR of 68% and a CR rate of 50%. CR rates in high-risk patient populations and in those who had received prior CAR-T therapy were consistent with the overall population of patients with relapsed or refractory follicular lymphoma. The median duration of response was 20.4 months and the median PFS was 11.8 months [110].

#### CAR T-cell therapies

CAR-T cell therapy involves collecting autologous or allogeneic T-cells and modifying them to produce CAR fusion proteins consisting of an antigen recognition moiety and T-cell signaling domain, then infusing the engineered T-cells back into the patient. Autologous CAR-T cell therapies directed against CD19 have been tested in patients with CLL and are reported to induce remission in these patients [10, 111]. CD19-specific CAR-T therapy resulted in remission for 8 of 14 (57%) patients with heavily pretreated, relapsed or refractory CLL in a pilot/phase 1 study [111] and in remission for 17 of 24 (71%) patients with CLL who had experienced treatment failure with anti-CD20 antibody, fludarabine, or bendamustine in a phase 1/2 trial [10]. In the phase 1/2 trial, CD19-specific CAR-T therapy showed efficacy in high-risk patients with CLL who did not respond to ibrutinib [10].

An ongoing, open-label, phase 1/2 trial of CD19-specific autologous CAR-T cells in patients with heavily pretreated, relapsed or refractory CLL (TRANSCEND CLL 004; NCT03331198) recently reported updated results for the phase 1 portion of the study [112]. All the patients had received prior ibrutinib treatment and half had failed both prior venetoclax and BTK inhibitor therapy. All reported manageable toxicities. The ORR was 82% and median PFS was 18 months at a median follow-up of 18 months; 50% of patients had maintained their responses at 18 months. The phase 2 portion of the study is currently enrolling. Preliminary results for an ongoing phase 1 trial of CD19-specific EGFRt/19-28z/4-1BBL “armored” CAR-T cells in patients with relapsed or refractory NHL or CLL (NCT03085173) have also been reported [113]. No severe cytokine release events were noted, and the complete response rate was 57% at the time of reporting. A phase 1 trial (ALLCAR19) of another CD19-specific CAR-T cell treatment (AUTO1) in patients with CLL and other B-cell lymphomas is also ongoing (NCT02935257). Recent preliminary data showed that 84% of patients with relapsed or refractory B-cell acute lymphocytic lymphoma who were treated with AUTO1 achieved MRD-negative CRs; 58% of patients remain on-study with continued MRD-negative remission (median follow-up: 12.2 months) [114].

Several clinical trials investigating CAR-T cell therapy in patients with CLL are currently recruiting patients. These include a phase 1/2a trial of CD20-specific CAR-T cells in patients with relapsed or refractory NHL or CLL (NCT04030195), a phase 1 trial of CD19-specific CAR-T cells in patients with CLL or diffuse large B-cell lymphoma (NCT03960840), a phase 1 trial of second- or third-generation CD19-specific CAR-T cells in patients with CLL, acute lymphocytic leukemia, or advanced B-cell NHL (NCT01853631), and a phase 1 trial of CD19/CD20-specific CAR-T cells in patients with relapsed or refractory CLL (NCT04007029).

While CAR-T therapy has shown remarkable efficacy in CLL to date, there can be substantial issues with treatment-related toxicity. Common AEs include cytokine-release syndrome, B-cell aplasia, neurotoxicity, and infection, all of which can be severe [115, 116]. Appropriate supportive care and management of toxicities are critical to the success of CAR-T therapy [115, 116]. In addition to toxicities, manufacture of these patient-specific therapies is costly and takes a significant amount of time, potentially limiting the number of patients who can be treated [117]. Research towards improving the CAR-T production platform is ongoing [117].

#### CAR-natural killer cell therapies

Natural killer (NK) cells can also be engineered to express CARs. It is anticipated that these cells may overcome both the toxic effects and manufacturing hurdles associated with CAR-T cell therapy [118]. NK cells play a key role in the innate immune system by targeting cancer cells and virally infected cells that have down-regulated human leukocyte antigen class I molecules or that express stress markers. NK cells from an allogeneic source can be administered without the need for full human leukocyte antigen matching, eliminating the requirement for production on a patient-by-patient basis as required by most CAR-T cell therapies.

Early-phase trials of CD19-specific CAR-NK treatment in patients with CLL and NHL showed responses without major toxicity [1118]. CD20-specific CAR-NK cells have also been investigated against primary CLL cells both in vitro and in the Daudi mouse model of Burkitt lymphoma [119, 120]. These cells demonstrated antitumor effects in both cases, demonstrating promise for further preclinical development.

## How we treat CLL

It is important to stay current on new clinical findings; this information will help inform appropriate clinical decisions. In general, we recommend determining treatment sequence based on individual patient characteristics. Current disease status, comorbidities, safety profiles of potential treatments, and patient preferences are important considerations. For example, if a patient with newly diagnosed CLL has a high disease burden, we would recommend against using venetoclax because of the increased risk of tumor lysis. Additionally, some patients, particularly during a pandemic, may not want to be hospitalized for monitoring of tumor lysis. In this case, we would be more likely to recommend oral therapy. If a patient has cardiac disease, we recommend treatment with acalabrutinib rather than venetoclax given the associated cardiac risks with the latter therapy. However, for patients who may prefer therapy over a fixed time period, venetoclax may still be the best option.

## Conclusion

BTK and BCL-2 inhibitors have replaced chemotherapy as the standard-of-care therapy for patients with CLL. In the era of chemotherapy, patients with *TP53* dysfunction were considered high risk because chemotherapy was ineffective. These patients are still considered to be at a high risk for progression while receiving kinase inhibitor therapy. BTK inhibitors with improved selectivity, such as the recently approved acalabrutinib, may provide patients with a treatment option having improved tolerability and efficacy compared with ibrutinib. It is hoped that the expanding array of BTK inhibitors in development will allow patients to switch to a different BTK inhibitor if resistance emerges because of acquired mutations.

Other targeted therapies, often combined with anti-CD20 therapy, are most useful in patients who have disease progression on a BTK inhibitor, or for whom BTK inhibitors are unsuitable. Combining anti-CD20 with BCL-2 inhibitors offers a short-term alternative to continuous BTK-inhibitor monotherapy. Other targeted therapy combinations such as BCL-2 and BTK inhibitors or triplet combinations that include anti-CD20 also show promise, particularly in high-risk patients. Emerging targeted therapies, such as CD3/CD20 bispecific antibodies, may provide further treatment options. In addition, cell-based therapies may prove able to fill the unmet need for effective treatment in patients who have progressed on BTK inhibitors or other targeted therapies, or who are intolerant of those therapies.

In addition to expanding the number of agents that can be used in the treatment of CLL, research addressing optimal treatment sequence, safety, and efficacy of combination therapies, and modifications of current treatment regimens such as intermittent sequence therapy, is needed to fill the gaps in current knowledge related to treatment decision-making and to address some of the current unmet needs in CLL therapy.

## Data Availability

Not applicable.

## Abbreviations

AE: Adverse event
BCL-2: B-cell leukemia/lymphoma 2
BiTE: Bispecific T cell engager
BLK: B lymphocyte kinase
BMX: Bone marrow tyrosine kinase gene in chromosome X
BTK: Bruton tyrosine kinase
CAR-T: Chimeric antigen receptor T-cell
CLL: Chronic lymphocytic leukemia
CLL-IPI: Chronic lymphocytic leukemia international prognostic index
CR: Complete remission
CRi: Incomplete count recovery
CTL: Cytotoxic T lymphocyte
EGFR: Epidermal growth factor receptor
HER2: Human EGFR-2
HER4: Human EGFR-4
HRQoL: Health-related quality of life
ITK: Interleukin-2–inducible T-cell kinase
iwCLL: International Workshop on chronic lymphocytic leukemia
JAK2: Janus kinase 2
MRD: Minimal residual disease
NHL: Non-Hodgkin lymphoma
NK: Natural killer
ORR: Overall response rate
OS: Overall survival
PFS: Progression-free survival
PI3Kδ: Phosphatidylinositol 4,5-bisphosphate 3-kinase catalytic subunit delta
SAE: Serious adverse event
SLL: Small lymphocytic lymphoma
uMRD: Undetectable minimal residual disease
WM: Wäldenstrom macroglobulinemia
